# Supplementary material for: Localization and functional characterization of the alternative oxidase in Naegleria
Source: J Eukaryot Microbiol. 2022 May 3;69(4):e12908. doi: 10.1111/jeu.12908 (PMC9540462; doi:10.1111/jeu.12908)
Supplement: Supplementary file 2 — Figure S1. Sequence alignments of AOX reveals conserved domains. Figure S2. Sequence alignments of AOX reveals conserved domains. Figure S3. Confocal microscopy demonstrating mitochondrial localization of AOX. Figure S4. High‐resolution real‐time respirometry raw trace data reveals cyanide resistant respiration. Table S2. Universally conserved amino acids of AOX and their roles. [file JEU-69-0-s002.pdf]

SUPPORTING INFORMATION

Localization and Functional Characterization of the Alternative Oxidase in *Naegleria* by Diego Cantoni, Ashley Osborne, Najwa Taib, Gary Thompson, Rubén Martín-Escolano, Eleanna Kazana, Elizabeth Edrich, Ian R. Brown, Simonetta Gribaldo, Campbell W Gourlay, and Anastasios D. Tsaousis

Figure S1. Sequence alignments of AOX reveals conserved domains
Maximum likelihood tree of AOX homologs in eukaryotes and bacteria (323 sequences, 148 amino acid positions). The tree was inferred with IQTREE using the LG+I+G4 model selected under the BIC criterion. Grey dots correspond to supports higher than 80%. The scale bar corresponds to the average number of substitutions per site.

Figure S2. Sequence alignments of AOX reveals conserved domains
To assess level of conservation between AOXs we aligned ngAOX, nfAOX and nlAOX amino acid sequences against other well characterized AOXs; *Trypanosoma brucei, Candida albicans, Arabidopsis thaliana, Cryptosporidium parvum* and*, Neurospora crassa.* We observed a considerable amount of conservation between all AOXs towards the middle and C-terminal end, where the presence of the alpha helical bundles and di-iron binding domains reside. * denotes key amino acids presented in Table below. † denotes a deviation in conserved amino acids of AOX

Figure S3. Confocal microscopy demonstrating mitochondrial localization of AOX.
Additional *N. gruberi* cells demonstrating localization of AOX in their mitochondria. *N. gruberi* cells were treated with mitotracker red prior to fixation and then probed with AOX antibodies (green). Nuclear marker and mtDNA staining is shown in blue (DAPI staining) Our confocal imaging reveals a high degree of co-localization between the mitotracker red signal and the green signal derived from immunoprobing AOX.

Figure S4. High resolution real-time respirometry raw trace data reveals cyanide resistant respiration.
Exemplar raw trace data used in **Figure 5** showing oxygen flux and oxygen concentration of *N. gruberi* cells in either M7 media (A) or PYNFH (B) media without glucose, following addition of respiration inhibitors, using an OROBOROS Oxygraph-2k with Clark polarographic oxygen electrodes.

Table S1. Excel file showing the AOX distribution across proteobacteria, other bacteria and eukaryotes (Tab 1, 2 and 3 respectively).

Table S2. Universally conserved amino acids of AOX and their roles. Comparison of amino acid residues between *Trypanosomas brucei brucei* AOX (trAOX) and *Naegleria gruberi* AOX (ngAOX), *Naegleria fowleri* AOX (nfAOX) and, *Naegleria lovaniensis* AOX (nlAOX)

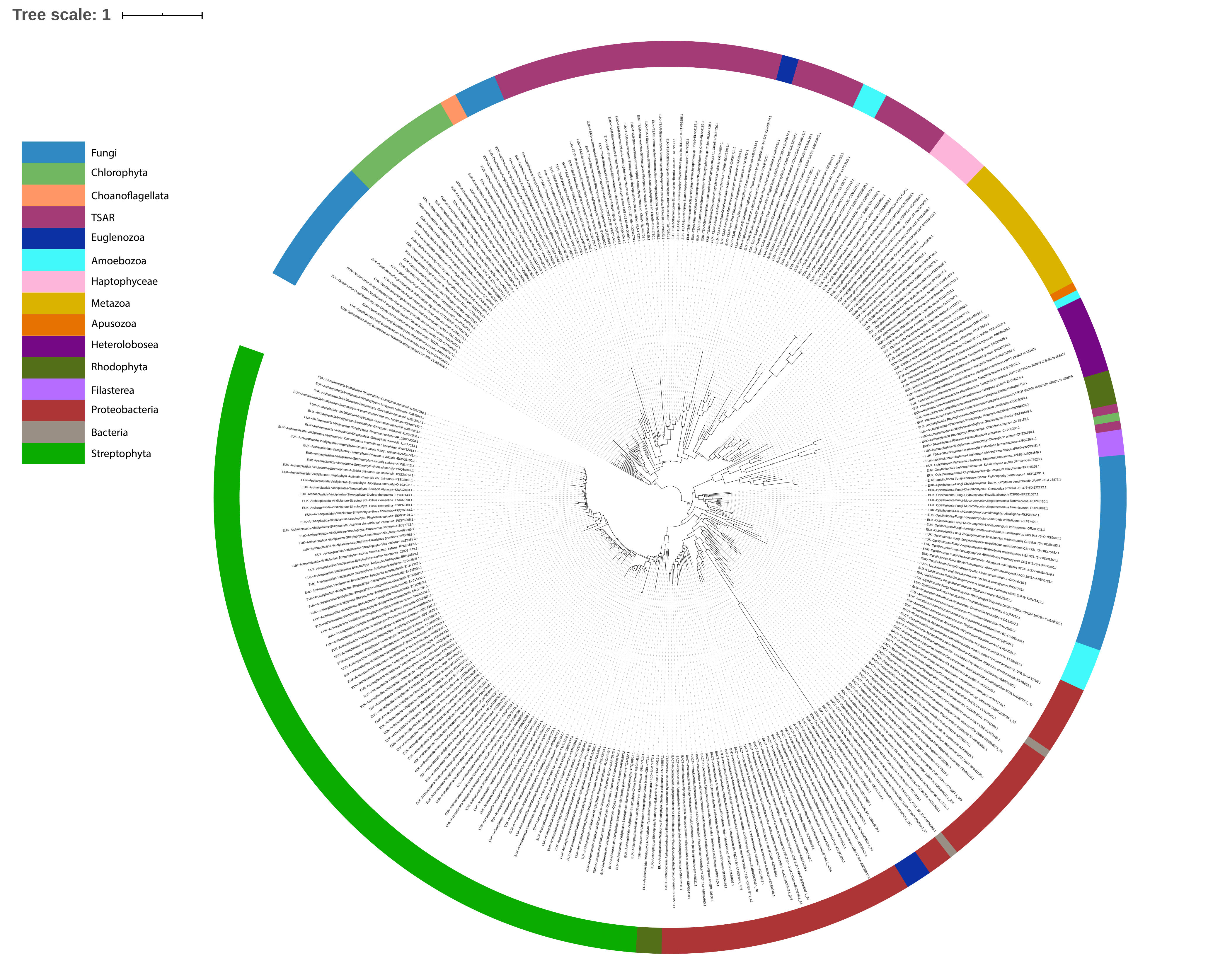

Tree scale: 1
Fungi
Chlorophyta
Choanoflagellata
TSAR
Euglenozoa
Amoebozoa
Haptophyceae
Metazoa
Apusozoa
Heterolobosea
Rhodophyta
Filasterea
Proteobacteria
Bacteria
Streptophyta

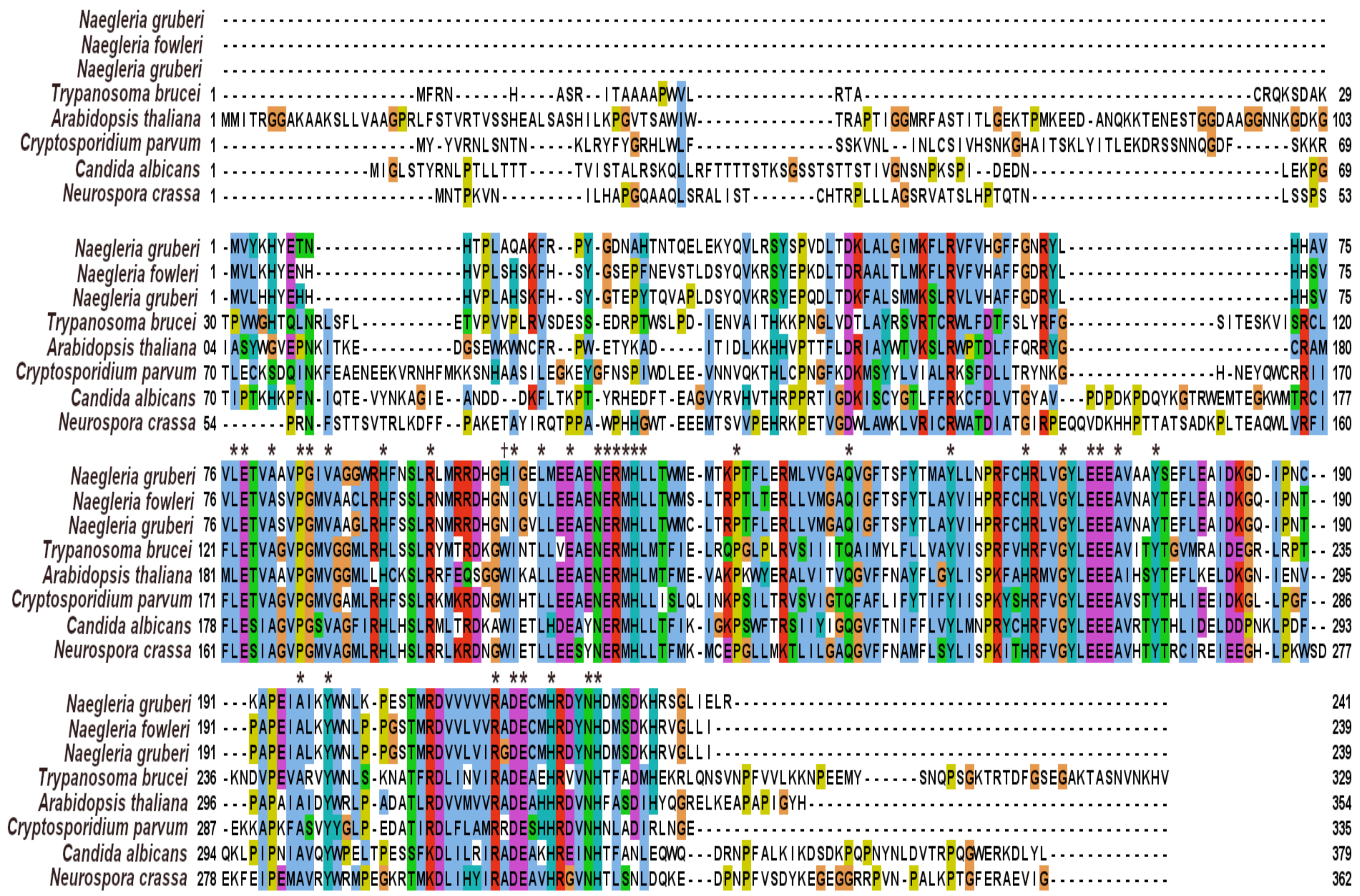

Naegleria gruberi --
Naegleria fowleri --
Naegleria gruberi --
Trypanosoma brucei 1 --------------------MFRN-----H---ASR--ITAAAAPWVL--------------RTA-------------------------------------CRQKSDAK 29
Arabidopsis thaliana 1 MMITRGGAKAAKSLLVAAGPRLFSTVRTVSSHEALSASHILKPGVTSAWIW--------------TRAPTIGGMRFASTITLGEKTPMKEED-ANQKKTENESTGGDAAGGNNKGDKG 103
Cryptosporidium parvum 1 --------------------MY-YVRNLSNTN----KLRYFYGRHLWLF--------------SSKVNL--INLCSIVHSNKGHAITSKLYITLEKDRSSNNQGDF-----SKKR 69
Candida albicans 1 ---------------MIGLSTYRNLPTLLTTT----TVISTALRSKQLLRFTTTTSTKSGSSTSTTSTIVGNSNPKSPI--DEDN--------------------------LEKPG 69
Neurospora crassa 1 ---------------------MNTPKVN---------ILHAPGQAAQLSRALIST------CHTRPLLLAGSRVATSLHPTQTN-------------------------LSSPS 53
Naegleria gruberi 1 -MVYKHYETN---------------HTPLAQAKFR--PY-GDNAHTNTQELEKYQVLRSYSPVDLTDKLALGIMKFLRVFVHGFFGNRYL--------------------------HHAV 75
Naegleria fowleri 1 -MVLKHYENH---------------HVPLSHSKFH--SY-GSEPFNEVSTLDSYQVKRSYEPKDLTDRAALTLMKFLRVFVHAFFGDRYL--------------------------HHSV 75
Naegleria gruberi 1 -MVLHHYEHH---------------HVPLAHSKFH--SY-GTEPYTQVAPLDSYQVKRSYEPQDLTDKFALSMMKSLRVLVHAFFGDRYL--------------------------HHSV 75
Trypanosoma brucei 30 TPWWGHTQLNRLSFL----------ETVPVVPLRVSDESS-EDRPTWSLPD-IENVAITHKKPNGLVDTLAYRSVRTCRWLFDTFSLYRFG--------------SITESKVISRCL 120
Arabidopsis thaliana 04 IASYWGVEPNKITKE----------DGSEWKWNCFR--PW-ETYKAD-----ITIDLKKHHVPTTFLDRIAYWTVKSLRWPTDLFFQRRYG--------------------------CRAM 180
Cryptosporidium parvum 70 TLECKSDQINKFEAENEEKVRNHFMKKSNHAASILEGKEYGFNSPIWDLEE-VNNVQKTHLCPNGFKDKMSYYLVIALRKSFDLLTRYNKG--------------H-NEYQWCRRII 170
Candida albicans 70 TIPTKHKPFN-IQTE-VYNKAGIE--ANDD--DKFLTKPT-YRHEDFT-EAGVYRVHVTHRPPRTIGDKISCYGTLFFRKCFDLVTGYAV---PDPDKPDQYKGTRWEMTEGKWMTRCI 177
Neurospora crassa 54 -------PRN-FSTTSVTRLKDFF--PAKETAYIRQTPPA-WPHHGWT-EEEMTSVVPEHRKPETVGDWLAWKLVRICRWATDIATGIRPEQQVDKHHPTTATSADKPLTEAQWLVRFI 160
** * ** * * * †* * * ****** * * * * * ** * *
Naegleria gruberi 76 VLETVAAVPGIVAGGWRHFNSLRLMRRDHGHIGELMEEAENERMHLLTWME-MTKPTFLERMLVVGAQVGFTSFYTMAYLLNPRFCHRLVGYLEEEAVAAYSEFLEAIDKGD-IPNC-- 190
Naegleria fowleri 76 VLETVASVPGMVAACLRHFSSLRNMRRDHGNIGVLLEEAENERMHLLTWMS-LTRPTLTERLLVMGAQIGFTSFYTLAYVIHPRFCHRLVGYLEEEAVNAYTEFLEAIDKGQ-IPNT-- 190
Naegleria gruberi 76 VLETVASVPGMVAAGLRHFSSLRNMRRDHGNIGVLLEEAENERMHLLTWMC-LTRPTFLERLLVMGAQIGFTSFYTLAYVIHPRFCHRLVGYLEEEAVNAYTEFLEAIDKGQ-IPNT-- 190
Trypanosoma brucei 121 FLETVAGVPGMVGGMLRHLSSLRYMTRDKGWINTLLVEAENERMHLMTFIE-LRQPGLPLRVSIIITQAIMYLFLLVAYVISPRFVHRFVGYLEEEAVITYTGVMRAIDEGR-LRPT-- 235
Arabidopsis thaliana 181 MLETVAAVPGMVGGMLLHCKSLRRFEQSGGWIKALLEEAENERMHLMTFME-VAKPKWYERALVITVQGVFFNAYFLGYLISPKFAHRMVGYLEEEAIHSYTEFLKELDKGN-IENV-- 295
Cryptosporidium parvum 171 FLETVAGVPGMVGAMLRHFSSLRKMKRDNGWIHTLLEEAENERMHLLISLQLINKPSILTRVSVIGTQFAFLIFYTIFYIISPKYSHRFVGYLEEEAVSTYTHLIEEIDKGL-LPGF-- 286
Candida albicans 178 FLESIAGVPGSVAGFIRHLHSLRMLTRDKAWIETLHDEAYNERMHLLTFIK-IGKPSWFTRSIIYIGQGVFTNIFFLVYLMNPRYCHRFVGYLEEEAVRTYTHLIDELDDPNKLPDF-- 293
Neurospora crassa 161 FLESIAGVPGMVAGMLRHLHSLRRLKRDNGWIETLLEESYNERMHLLTFMK-MCEPGLLMKTLILGAQGVFFNAMFLSYLISPKITHRFVGYLEEEAVHTYTRCIREIEEGH-LPKWSD 277
* * * ** * **
Naegleria gruberi 191 ---KAPEIAIKYWNLK-PESTMRDVVVVVRADECMHRDYNHDMSDKHRSGLIELR--- 241
Naegleria fowleri 191 ---PAPEIALKYWNLP-PGSTMRDVVLVVRADECMHRDYNHDMSDKHRVGLLI-- 239
Naegleria gruberi 191 ---PAPEIALKYWNLP-PGSTMRDVVLVIRGDECMHRDYNHDMSDKHRVGLLI-- 239
Trypanosoma brucei 236 -KNDVPEVARVYWNLS-KNATFRDLINVIRADEAEHRVVNHTFADMHEKRLQNSVNPFVVLKKNPEEMY------SNQPSGKTRTDFGSEGAKTASNVNKHV 329
Arabidopsis thaliana 296 ---PAPAIAIDYWRLP-ADATLRDVVMVVRADEAHHRDVNHFASDIHYQGRELKEAPAPIGYH--- 354
Cryptosporidium parvum 287 -EKKAPKFASVYYGLP-EDATIRDLFLAMRRDESHHRDVNHNLADIRLNGE-- 335
Candida albicans 294 QKLPIPNIAVQYWPELTPESSFKDLILRIRADEAKHREINHTFANLEQWQ---DRNPFALKIKDSDKPQPNYNLDVTRPQGWERKDLYL------------ 379
Neurospora crassa 278 EKFEIPEMAVRYWRMPEGKRTMKDLIHYIRADEAVHRGVNHTLSNLDQKE---DPNPFVSDYKEGEGGRRPVN-PALKPTGFERAEVIG------------ 362

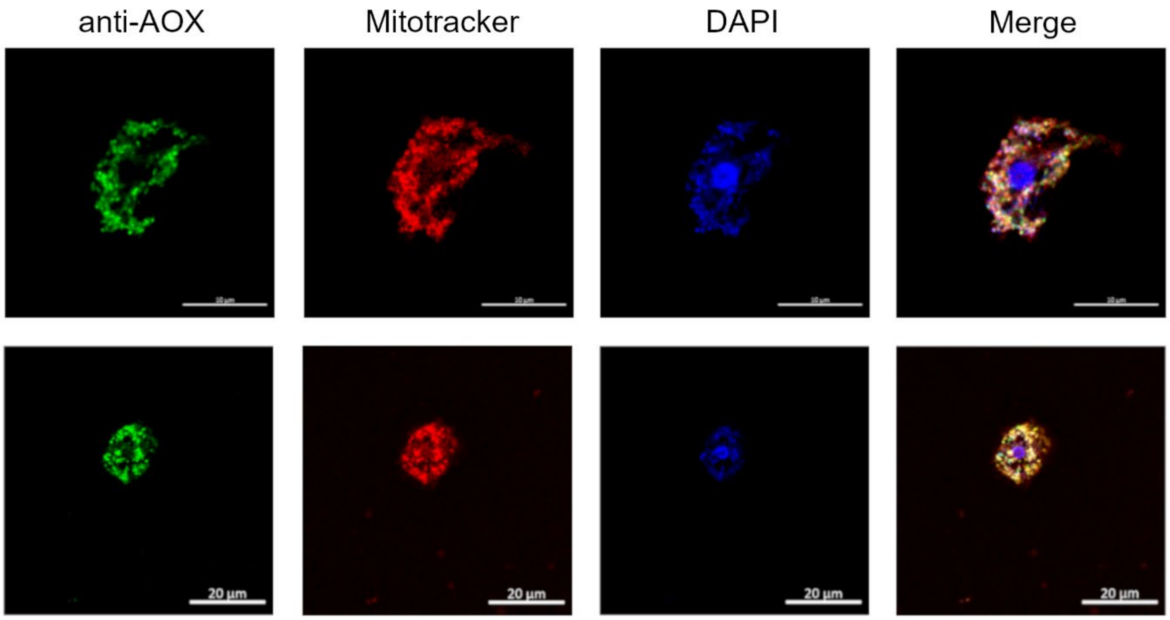
anti-AOX
Mitotracker
DAPI
Merge
20 µm
20 µm
20 µm
20 µm

(A)

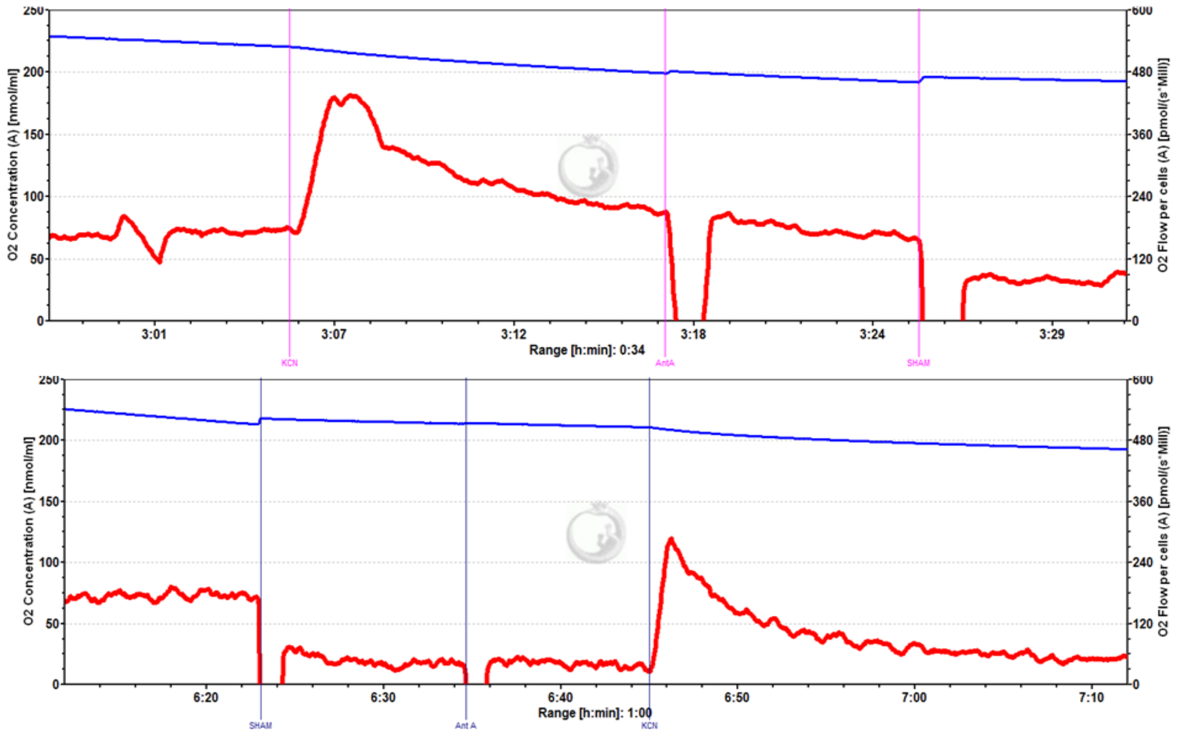

O2 Concentration (A) [nmol/ml]
O2 Flow per cells (A) [pmol/(s*Mill)]
Range [h:min]: 0:34
KCN
AntA
SHAM
3:01
3:07
3:12
3:18
3:24
3:29
Range [h:min]: 1:00
SHAM
Ant A
KCN
6:20
6:30
6:40
6:50
7:00
7:10

(B)

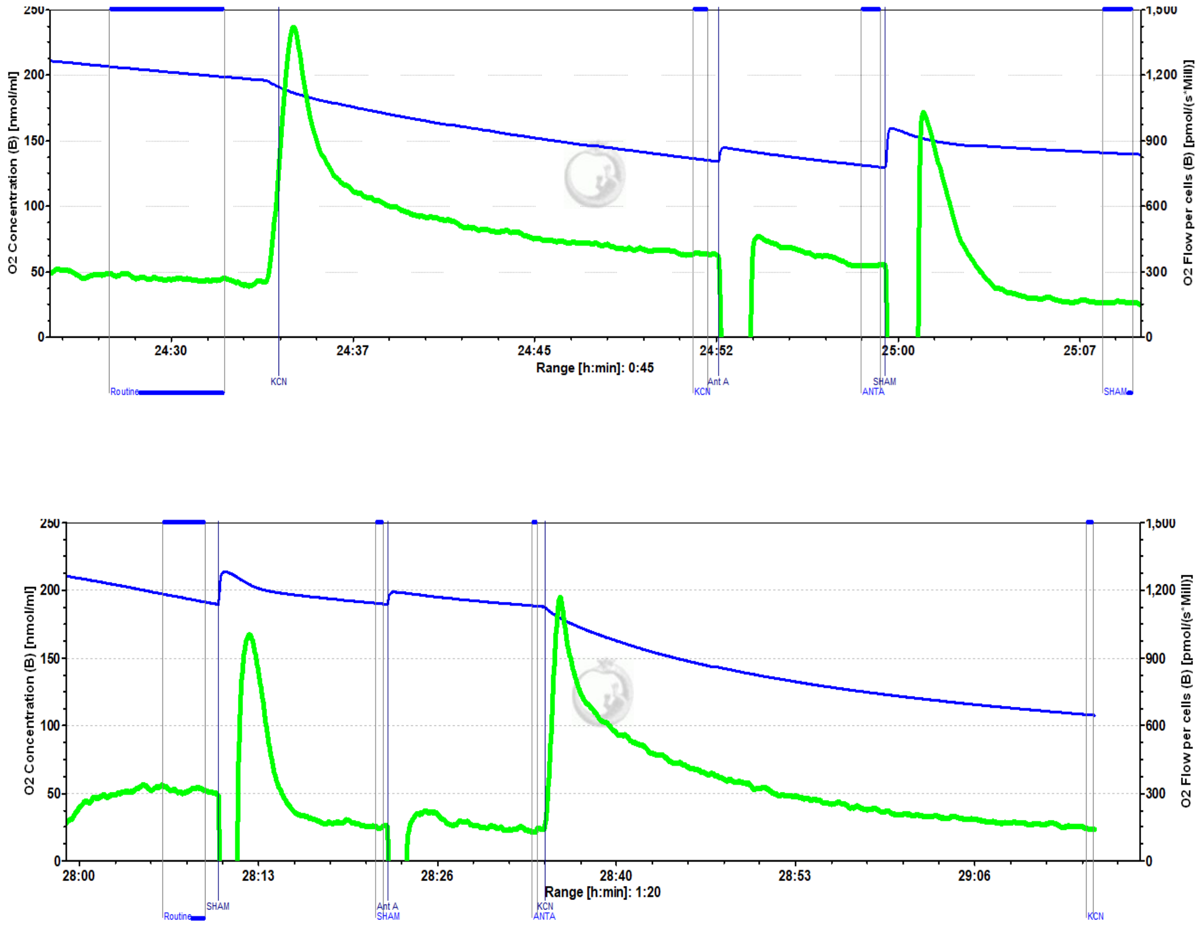

O2 Concentration (B) [nmol/ml]
O2 Flow per cells (B) [pmol/(s*Mill)]
Range [h:min]: 0:45
Routine
KCN
Ant A
SHAM
ANTA
24:30
24:37
24:45
24:52
25:00
25:07
Range [h:min]: 1:20
28:00
28:13
28:26
28:40
28:53
29:06

Table S2. Universally conserved amino acids of AOX and their roles. Comparison of amino acid residues between *Trypanosomas brucei brucei* AOX (trAOX) and *Naegleria gruberi* AOX (ngAOX), *Naegleria fowleri* AOX (nfAOX) and, *Naegleria lovaniensis* AOX (nlAOX)

**tbAOX numbering**	**ngAOX numbering**	**nfAOX numbering**	**nlAOX numbering**	**Role**
Leu-122	Leu-77	Leu-77	Leu-77	Substrate-binding channels 1 and 2
Glu-123†	Glu-78	Glu-78	Glu-78	Fe-Fe Ligand; substrate binding channels 1 and 2
Ala-126	Ala-81	Ala-81	Ala-81	Substrate-binding channels 1 and 2
Pro-129	Pro-84	Pro-84	Pro-84	Substrate-binding channels 2
Gly-130	Gly-85	Gly-85	Gly-85	Forms kink in helix α2
Val-132	Val-87	Val-87	Val-87	Hydrophobic interaction with helix α6
His-138	His-93	His-93	His-93	Membrane-binding region/dimer interface
Arg-143	Arg-98	Arg-98	Arg-98	Membrane-binding region/dimer interface
Trp-151	*His-106*	*Asn-106*	*Asn-106*	*Hydrophobic interaction with helix α6*
Iso-152	Iso-107	Iso-107	Iso-107	Dimer interface
Leu-155	Leu-110	Leu-110	Leu-110	Dimer interface
Glu-158	Glu-113	Glu-113	Glu-113	Substrate-binding channels 1 and 2
Asn-161†	Asn-116	Asn-116	Asn-116	Secondary ligation sphere; hydrogen bond network
Glu-162†	Glu-117	Glu-117	Glu-117	Fe-Fe Ligand
Arg-163	Arg-118	Arg-118	Arg-118	Membrane-binding region
Met-164	Met-119	Met-119	Met-119	Dimer interface; interaction with N-terminal arm
His-165	His-120	His-120	His-120	Fe-Fe ligand
Leu-166	Leu-121	Leu-121	Leu-121	Dimer interface
Pro-175	Pro-130	Pro-130	Pro-130	Dimer interface
Gln-187	Gln-142	Gln-142	Gln-142	Dimer interface
Tyr-198†	Tyr-153	Tyr-153	Tyr-153	Dimer interface; hydrogen bonds to His-206 (tbAOX)
His-206	His-161	His-161	His-161	Membrane-binding region
Gly-210	Gly-165	Gly-165	Gly-165	Forms kink in helix α5
Glu-213†	Glu-168	Glu-168	Glu-168	Fe-Fe ligand
Glu-214	Glu-169	Glu-169	Glu-169	Interacts with N-terminal arm
Ala-216	Ala-171	Ala-171	Ala-171	Substrate-binding channel 2
Tyr-220†	Tyr-175	Tyr-175†	Tyr-175	Catalytic cycle
Ala-243	Ala-196	Ala-196	Ala-196	Hydrophobic interaction with helix α3
Tyr-246†	Tyr-199	Tyr-199	Tyr-199	Secondary ligation sphere; hydrogen bond network
Arg-263	Arg-216	Arg-216	Arg-216	Interaction with helix α5 and N-terminal arm
Asp-265†	Asp-218	Asp-218	Asp-218	Secondary ligation sphere; hydrogen bond network
Glu-266	Glu-219	Glu-219	Glu-219	Fe-Fe ligand
His-269	His-222	His-222	His-222	Fe-Fe ligand
Asn-273	Asn-226	Asn-226	Asn-226	Interaction with helix α5
His-274	His-227	His-227	His-227	Interaction with helix α5

†Denotes universally conserved residues of AOX
